# Triangular Screw Placement to Treat Dysmorphic Sacral Fragility Fractures in Osteoporotic Bone Results in an Equivalent Stability to Cement-Augmented Sacroiliac Screws—A Biomechanical Cadaver Study

**DOI:** 10.3390/jcm14051497

**Published:** 2025-02-24

**Authors:** Isabel Graul, Ivan Marintschev, Antonius Pizanis, Marcel Orth, Mario Kaiser, Tim Pohlemann, Working Group on Pelvic Fractures of The German Trauma Society, Tobias Fritz

**Affiliations:** 1 Jena University Hospital, Department of Trauma, Hand and Reconstructive Surgery, Friedrich Schiller University Jena, 07740 Jena, Germany; 2 Department of Trauma, Orthopedics and Spine Surgery, Catholic Hospital “St. Johann Nepomuk”, 99097 Erfurt, Germany; ivan.marintschev@kkh-erfurt.de; 3 Department for Trauma, Hand and Reconstructive Surgery, Saarland University Medical Center, 66421 Homburg, Germany; antonius.pizanis@uks.eu (A.P.); marcel.orth@uks.eu (M.O.); tim.pohlemann@uks.eu (T.P.); tobias.fritz@uks.eu (T.F.); 4 Jenoptik GmbH, 07745 Jena, Germany; mario.kaiser83@icloud.com; 5 German Society for Trauma Surgery, 10623 Berlin, Germany; andreas.hoech@uni-leipzig.de

**Keywords:** sacral insufficiency fractures, operative treatment, sacroiliacal screws, transsacral, oblique

## Abstract

**Background:** Sacroiliac screw fixation in elderly patients with pelvic fractures remains a challenging procedure for stabilization due to impaired bone quality. To improve it, we investigated the biomechanical properties of combined oblique sacroiliac and transiliosacral screw stabilization versus the additional cement augmentation of this construct in a cadaver model of osteoporotic bone, specifically with respect to the maximal force stability and fracture-site motion in the displacement and rotation of fragments. **Methods***:* Standardized complete sacral fractures with intact posterior ligaments were created in osteoporotic cadaver pelvises and stabilized with a triangle of two oblique sacroiliac screws from each side with an additional transiliosacral screw in S1 (*n* = 5) and using the same pelvises with additional cement augmentation (*n* = 5). A short cyclic loading protocol was applied, increasing the axial force up to 125 N. Sacral fracture-site motion in displacement and rotation of the fragments was measured by optical motion tracking. **Results:** A maximum force of 65N +/− 12.2 N was achieved using the triangular screw stabilization of the sacrum. Cement augmentation did not provide any significant gain in maximum force (70 N +/− 29.2 N). Only low fragment displacement was observed (2.6 +/− 1.5 mm) and fragment rotation (1.3 +/− 1.2°) without increased stability (3.0 +/− 1.5 mm; *p* = 0.799; 1.7 +/− 0.4°; *p* = 0.919) following the cement augmentation. **Conclusions:** Triangular stabilization using two obliques and an additional transiliosacral screw provides sufficient primary stability of the sacrum. Still, the stability achieved seems very low, considering the forces acting in this area. However, additional cement augmentation did not increase the stability of the sacrum. Given its lack of beneficial abilities, it should be used carefully, due to related complications such as cement leakage or nerve irritation. Improving the surgical methods used to stabilize the posterior pelvic ring will be a topic for future research.

## 1. Introduction

The incidence of pelvic fractures in the aging population has risen significantly, with several studies reporting an increase in low-energy fractures, especially among the elderly [1,2,3] (estimated that this number will rise 2.4 times by 2030. These fractures result from decreased mineral bone density and low-energy injury patterns (e.g., ground-level falls) [4], with a yearly incidence up to 92/100,000 in the population aged over 65 years, and up to 446/100,000 for the population aged over 85 years [5]. These fractures often result in immobilization of the patients and persistent pain. Additionally, the literature frequently describes fracture progression and secondary displacement, often leading to immobilization and increased pain levels [6,7].

Most cases of fragility fractures can be treated conservatively with early mobilization and adequate pain medication, which is the primary goal of this therapy. However, if conservative treatment fails to alleviate the patient’s pain or the fracture displacement progresses rapidly, surgical stabilization is recommended [8,9,10,11]. Therefore, numerous techniques have been described for the fixation of the posterior pelvic ring. Iliosacral screw fixation of the first or second sacral body is well-established and a preferred method for stabilization [8,11,12,13], with technical variants regarding screw angulation, which can be placed horizontally, oblique [14], or even transiliosacral through the other side [15].

However, high rates of screw loosening, migration, or cut-out can be seen during the postoperative follow-up [10,16,17,18,19]. In particular, the reduced bone stock of the sacrum in elderly or osteoporotic patients [20,21,22,23,24] results in screw loosening in up to 20% of all cases [8]. Therefore, the placement of multiple or transiliosacral screws seems to reduce this complication rate [8]. Many studies focused on the use of cement augmentation of sacroiliac screws to improve screw fixation and add more stability to the construct [25]. Suero et al. showed that a single cemented screw provides the same biomechanical stability as non-augmented double screws in the treatment of sacral fractures [25]. Although cement augmentation increased the anchorage at the distal screw end, current studies show that it cannot prevent the screws from loosening [9]. Furthermore, the use of cement can result in complications such as cement leakage into the neuroforamina and damage to the neurological structures. In the current study, we aimed to analyze the biomechanical stability of a bilateral sacral fragility fracture, according to an FFP IVb or OF4 fracture, which was stabilized by two oblique and one transiliosacral screw in the S1 body with and without cement augmentation. We hypothesized that cement augmentation would increase the stability of the construct.

## 2. Methods

### 2.1. Specimen

Approval of the institution’s ethics committee (Protocol Nr. 131/21) prior to the start of this study was obtained. Cadavers were included after body donors gave written informed consent during their lifetimes. A total of 5 adult human cadaver pelvis specimens (complete pelvic ring including the fourth and fifth lumbar vertebrae) from the Institute of Anatomy were obtained with consent from the donors for medical teaching and research. The pelvises were fresh frozen at −20 °C and thawed 18 h before testing. The mean age of the donors was 78.1 years (±18.3) and they were equally distributed between female (*n* = 3) and male (*n* = 2).

Computed tomography (CT) scans were performed on all specimens using a clinical 128-slice Spiral CT scanner (Siemens Definition Edge, Siemens, Erlangen, Germany). Each scan was conducted with a tube voltage of 120 kV and a tube current of 300 mAs. Image reconstruction was carried out using the scanner’s manufacturer-provided reconstruction kernels. A bone window was reconstructed with a dedicated kernel (I70h) at a slice thickness of 0.5 mm, while a soft-tissue window was reconstructed using another dedicated kernel (I41s) at a slice thickness of 1 mm. All CT images were oriented in the transverse plane, saved in DICOM (Digital Imaging and Communication in Medicine) format, and transferred to a workstation running Horos™ v3.3.5 for further analysis.

Bone density was assessed by measuring Hounsfield units (HU) in S1 within circular regions of interest (ROIs) positioned midaxially and midsagittally in the S1 body [26]. The mean bone density of the pelvises was 106.6 ± 44.2 HU, measured in the S1 body. All specimens exhibited a bone density below 202 HU in the S1 body, classifying them as osteoporotic (Table 1).

Before testing, all soft tissues were removed from the pelvises, except for the anterior and posterior sacroiliac ligaments, which remained intact. To simulate an instable sacral bilateral fracture, a L-shaped wooden osteotomy saw gauge template was placed medial to the posterior iliac spine and used in all specimens. Hereafter, using the saw gauge, an osteotomy 1 cm medial to the posterior iliac spine was performed in the sacral ala bilateral region of each pelvis with an oscillating saw. Each osteotomy was 2 mm wide and involved the entire length of the sacrum; the posterior cortex and the posterior sacroiliac ligaments were left intact [27]. This fracture pattern mimics an FFP-IVb/OF4 fracture [7,28].

To achieve anterior stability of the pelvic ring, so an anterior instability would not interfere with the results for the posterior in this study, the symphysis was stabilized using a 4-hole interlocking symphysis plate (Synthes, Oberdorf, Switzerland) in all pelvises according to previous studies [29,30,31]

### 2.2. Sensors

To monitor the movement of fracture fragments, an optical measuring system with four cameras (Prime 13^®^, Optitrack, Corvallis, OR, USA) was employed. This system, with a resolution of 1280 × 1024 pixels, achieves an accuracy of 0.2 mm by attaching at least two optical markers to each fragment. The system-specific software (Motive 2.1^®^, version 2, Optitrack, Corvallis, OR, USA) processed the measurements into a corresponding coordinate system and a 3D movement model. For analysis, three primary fragments were identified: the “left sacral fragment”, “central fragment”, and “right sacral fragment”. Each was marked with 2–3 optical markers using a standardized template to ensure reproducible positioning. Mathematical calculations determined the relative movement of these fragments in three spatial planes [29,31]. The maximum fracture displacement was defined as the greatest fragment separation under loading conditions.

### 2.3. Experimental Setup

In this study, we investigated the following 2 groups (*n* = 5).

**Figure 1:**
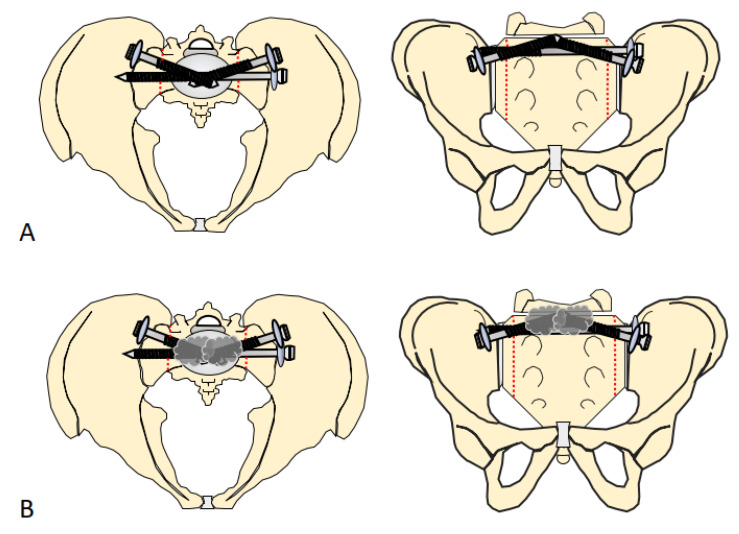
(**A**) Schematic drawing of the triangular fixation of the sacrum. Two oblique screws in the S1 vertebra and a transiliosacral screw in the S2 vertebra. (**B**) Schematic drawing of the additional cement augmentation of the oblique screws in the S1 vertebra. Dotted red line represents the fracture-lines.

Group 1: In this group, we stabilized the sacral fracture using two partially threaded sacroiliac screws from both sides, oblique inverted (cannulated, partial threaded steel screw 7.3 mm, DePuy Synthes, Raynham, MA, USA), and a fully threaded transiliosacral screw (cannulated, fully threaded steel screw 7.3 mm, DePuy Synthes, Raynham, MA, USA), which led to a total of 6 cortical index osteosynthesis procedures of groups 1 and 2 together (Figure 1A).

Group 2: In this group, additional cement augmentation with high-viscosity PMMA (Traumacem^TM^ V+, DePuy International Co., Ltd., Leeds, UK) was applied. To achieve that, the oblique sacroiliac screws were removed partially, and then the cement was applied and the screws were fastened immediately [32] (Figure 1B).

Sacroiliac screw osteosynthesis was conducted under fluoroscopic control using a standard inlet/outlet fluoroscopic technique [33].

**Figure 2:**
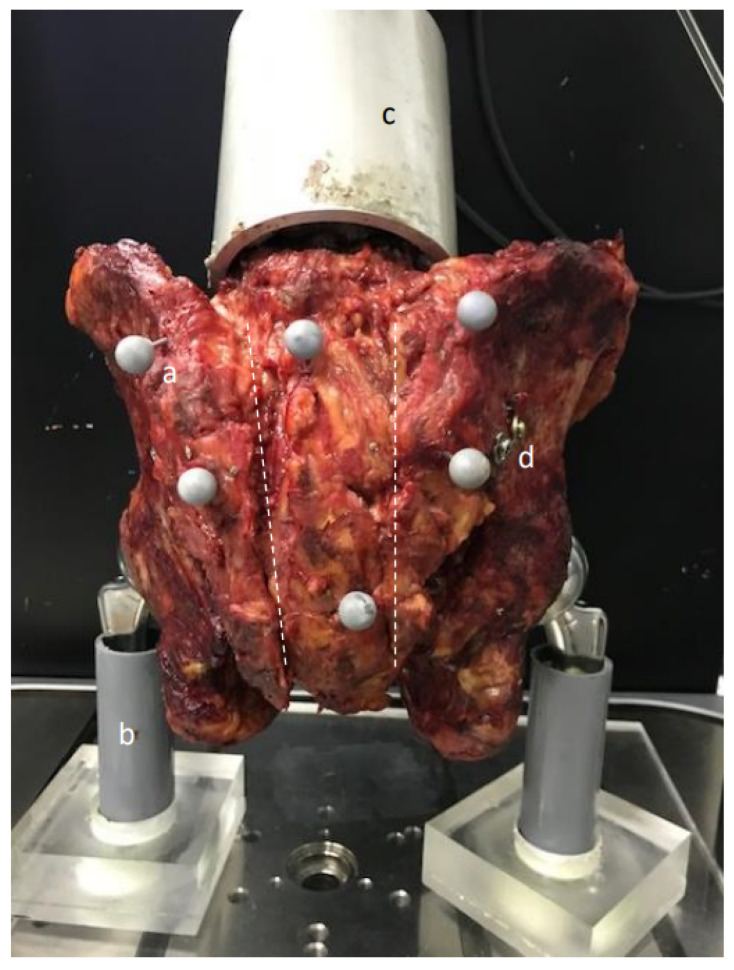
Visualization of the biomechanical test setup with supplied fracture and inserted optical markers. Dotted white line represents the fracture-lines. (**a**) optical marker, (**b**) holder on the base plate, (**c**) mounting to the universal testing machine, (**d**) visible screw head.

To allow a controlled load distribution, all pelvises were tested by a simulated bilateral-leg stance model as described in previous studies [34]. Before mounting the pelvis in the universal testing machine (Instron ElectroPuls E10000, Instron, Norwood, MA, USA), 6 optical markers (Optitrack, Corvallis, OR, USA) were added to the fracture fragments (2 for each fragment) as recommended by the manufacturer (Figure 2). To simulate a bilateral stance as it occurs during walking, bipolar cephalic hip prostheses (48 mm, Zimmer Inc., Warsaw, IN, USA) were used and articulated with both acetabula. Both prostheses were set up on ball-bearing slide plates, which allowed independent coronal movement, and thus extrinsic stability of the mounted pelvis. After positioning the pelvises, axial loading was applied with a ceramic hip prosthesis head (28 mm, Zimmer Inc., Warsaw, IN, USA) that was articulated at 45° to the proximal sacrum in a fitting cement cup, which was based on a custom-built mount. The mount was fixed on the L5 vertebra using Technovit (Heraeus Kulzer, Wehrheim, Germany).

After mounting the pelvises in the universal testing machine, a baseline pre-force of 25 N was applied. Each cycle consisted of a maximum load and a relief plateau, both held for 10 s. The first two loading cycles served as setting cycles, after which fracture displacement was measured over the following five cycles. The applied force was incrementally increased by 25 N steps, starting from the initial 25 N, until a fracture displacement of 2 mm was reached, to prevent complete specimen failure. Osteosynthesis failure was defined as either a fracture displacement of 2 mm or implant breakage/pelvic fracture.

The maximum load of 200 N was determined based on the existing literature, which yielded comparable results [25,35].

#### Data Acquisition, Processing, and Statistical Analysis

Spatial instability and movement were recorded using an optical measurement system (Prime 13^®^, Optitrack, Corvallis, OR, USA), with marker pairs placed in standardized positions on each pelvis. Data analysis was conducted using the manufacturer’s software (Motive 2.1^®^, Optitrack, Corvallis, OR, USA). Statistical analysis was performed using SPSS (version 22.0, Chicago, IL, USA) and SigmaPlot (SigmaPlot 13.0; Systat Software Inc., San José, CA, USA). The Wilcoxon test was used to compare groups, and mean values with standard deviations were calculated for continuous variables. A *p*-value of <0.05 was considered statistically significant.

## 3. Results

**Figure 3:**
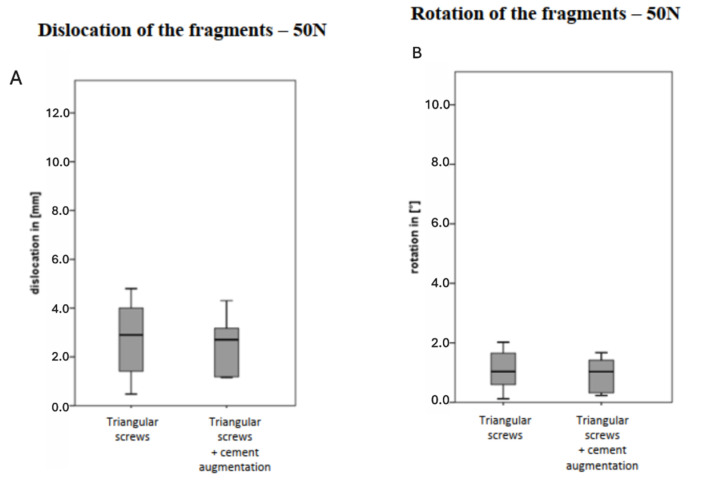
(**A**) Displacement of the sacral fragments from the central fragment in the different osteosynthesis procedures. (**B**) Rotation of the sacral fragments in relation to the central fragment in the different osteosynthesis procedures.

A mean displacement of 6.5 +/− 2.7 mm (*p* < 0.05), and rotational pivoting of 4.4 +/− 2.4°; (*p* < 0.05) of the sacrum were detected for the triangular stabilization group (vs. baseline load). The additional cement augmentation resulted in a tendency for lower movement, but without significantly increasing the stability, when compared to the triangular stabilization, respectively, 3.0 +/− 1.5 mm (*p* < 0.05) for translation and 1.7 +/− 0.4°; (*p* < 0.05) for rotation (Figure 3A,B).

The load to failure reached 65 N +/− 12.2 N in the triangular group. The additional cement augmentation allowed a slightly higher load to failure, but without significance (70 N +/− 29.2 N, *p* > 0.05 vs. triangular screws). The maximum load to failure values are shown in Table 1.

## 4. Discussion

The treatment of fractures in the geriatric population requires an improvement of currently existing options. The results of the present study show that stable fixation using triangular iliosacral screws can be achieved; however, additive cement augmentation did not increase the stability of the triangular screw stabilization technique for the sacrum. Previous studies have reported a prevalence of dysmorphic S1 vertebrae ranging from 14% to 64% [36,37,38]. Wagner et al. also found that in an additional 25% of analyzed cases, the safe S1 corridor was deemed critical [37,38]. These findings highlight the need to explore alternative fixation methods to ensure sacral stability. Based on these results, we propose that alternative fixation methods should be considered to achieve stability of the sacrum. It has been claimed that longer iliosacral screws provide a more stable construct, because they have greater resistance to buckling and are more resistant to vertical shear loading [15]. Griffin et al. used a range of different screw lengths to analyze their impact on the failure rate; however, the only significant risk factor for fixation failure was the vertical sacral fracture and not the length of the iliosacral screw [16]. Also, Tornetta et al. found that a construct with a standard iliosacral screw combined with a long iliosacral screw crossing the contralateral sacroiliac joint did not increase the results compared to a construct with two iliosacral screws [10,16]. To further investigate these findings, this present biomechanical study was designed to analyze the stability of iliosacral screws in osteoporotic bone without and with cement augmentation. For this study, only cadavers with osteoporotic bone quality were selected to simulate unstable fractures of the geriatric patient. The bone quality was analyzed in HU in a region of interest in the S1 vertebra as described in the literature [26] and all pelvises showed an osteoporotic bone quality [24]. The groups were matched prior to testing for bone density, age, and sex. Although the sample size with five pelvises was small, this is in line with studies responding to similar scientific questions [39,40].

The fractures were created by an osteotomy according to previous studies [41] using the saw gauge, and the fracture pattern created was similar in all pelvises of this study. However, the fracture pattern created only mimicked fragility fractures of the FFP type IVb/OF4 without a horizontal fracture line, which are considered the most unstable fragility fractures, often resulting in prolonged episodes of pain [6]. Additionally, the symphysis was stabilized, to increase the stability of the biomechanical testing setup, as in previous studies [29,30,31].

For the biomechanical testing, a bilateral stance model was used [34,42]. Because the axial loading force was applied directly to the sacrum to analyze the stability of the osteosynthesis, an alternating single-leg stance model, which was also considered, was not required [29,31,43]. Screw configurations were tested sequentially in the same specimens to allow a direct comparison between the stabilization without cement augmentation and after augmentation with cement, even in a small sample of pelvises. This cumulative loading may influence the stiffness of the cadaveric model, but such testing sequences are commonly used in comparable vitro studies [41]. In this study, a maximum load of 125 N was achieved, due to the completely unstable bilateral transalar osteotomies and the exclusive use of osteoporotic cadavers. These results are in line with the described maximum loading forces in the literature [41,44]. Additionally, the displacement of >2 mm was considered a failure in this study, whilst other authors allowed a larger scale of displacement during their studies [41]. All pelvises in this study achieved sufficient stability after the osteosynthesis using the triangular iliosacral screws. The combination of oblique iliosacral screws in S1 and a transiliosacral screw in S2 was analyzed in the present study. Hasenboehler et al. showed higher body means for S2 in patients with dysmorphic sacra [45]. Due to the osteoporotic bone density, the construct remained fragile. To increase the stability, the use of cement augmentation seems beneficial [9,18,19,25,32,46]. In their study, Frey et al. showed that even a sacroplasty is beneficial after ten years [47]. Höch et al. also found reduced pain after cement-augmented sacroiliac screws for fragility fractures in a 1-year follow-up [41]. However, in biomechanical studies, similar to our model, no improvement in stiffness in the sacroiliac joint compared to a single non-augmented screw was found [18,41]. Ronchi et al. highlighted the complexity of fragility fractures of the pelvis and the resulting biological damage and physical impairment [48,49].

Additional to surgical therapy, controlled loading–unloading cycles through physical therapy and weight-bearing exercises to enhance bone strength should be considered (https://theros.org.uk/information-and-support/bone-health/exercise-for-bones, accessed on 15 January 2025). The results of the present study show that additive cement augmentation did not increase the stability of the osteosynthesis of the sacrum. Hence, the risk of additional complications such as cement leakage should be considered carefully in view of the lack of stability improvement produced by cement augmentation when compared to triangular oblique/transiliosacral screw fixation without cement.

## 5. Limitations

There are several limitations to this study. Biomechanical testing using fresh frozen specimens offers the ability to directly compare osteosynthesis techniques in a controlled setting. However, variations in bone and soft-tissue quality, as well as possible small variations in screw placement, may influence the results and may prevent a direct translation of the findings to in vivo findings. Furthermore, the number of cases in this study with isolated oblique and transiliosacral screws is low; however, when considering the highly homogenous osteoporotic bones sampled, the small sample size can be regarded as reliable.

## 6. Conclusions

A triangular-shaped fixation technique for the sacrum results in a comparable stability to that with cement augmentation of the screws, without the risk of cement leakage. As such, we found that the latter did not improve primary stability in this biomechanical study. However, further research on surgical techniques to stabilize fragility fractures of the sacrum must be conducted.

## Figures and Tables

**Table 1:** All maximum forces achieved [in N] for the 4 osteosynthesis groups and bone density [in HU].

	Without Augmentation	With Cement Augmentation	Bone Density in HU
1	0	75	77
2	75	50	107
3	75	125	192
4	0	50	82
5	50	50	75

## Data Availability

Data will be made available on request to the contributing author.
